# Evasion of I Interferon-Mediated Innate Immunity by Pseudorabies Virus

**DOI:** 10.3389/fmicb.2021.801257

**Published:** 2021-12-14

**Authors:** Rui Zhang, Jun Tang

**Affiliations:** College of Veterinary Medicine, China Agricultural University, Beijing, China

**Keywords:** PRV, IFN-I, antiviral innate immunity, immune evasion, JAK-STAT pathway

## Abstract

Type I interferon (IFN-I) mediated innate immunity serves as the first line of host defense against viral infection, ranging from IFN-I production upon viral detection, IFN-I triggered signaling pathway that induces antiviral gene transcription the antiviral effects of IFN-I induced gene products. During coevolution, herpesviruses have developed multiple countermeasures to inhibit the various steps involved to evade the IFN response. This mini-review focuses on the strategies used by the alphaherpesvirus Pseudorabies virus (PRV) to antagonize IFN-I mediated innate immunity, with a particular emphasis on the mechanisms inhibiting IFN-I induced gene transcription through the JAK-STAT pathway. The knowledge obtained from PRV enriches the current understanding of the alphaherpesviral immune evasion mechanisms and provides insight into the vaccine development for PRV control.

## Introduction

Pseudorabies virus (PRV) is a swine alphaherpesvirus closely related to the human herpes simplex virus type 1 (HSV-1) and varicella-zoster virus. PRV infects a broad host range of mammals. PRV infection primarily causes an acute lytic infection in its natural host, the adult pig, characterized by respiratory distress and reproductive failure while resulting in neurological symptoms and high mortality in the newborn piglets and non-natural hosts (Sun et al., 2016). Although PRV has been eradicated from domesticated pigs in North America and many European countries due to the frequent testing and extensive vaccination, it remains one of the most important swine diseases in many countries, including China. In particular, antigenically different PRV variants have emerged in China since 2011 (An et al., 2013; Wang et al., 2017), making PRV more difficult to control with vaccination. Currently, it was reported that PRV infection might also be a potential threat to humans, arousing the serious concern of epidemiologists and virologists (Ai et al., 2018; Yang et al., 2019; Fan et al., 2020; Liu et al., 2020; Wang D. et al., 2020). Like other alphaherpesviruses, PRV usually establishes lifelong latent infection in the host’s peripheral nervous system and has served as a useful model for studying herpesvirus biology and the host’s innate immune response (Brittle et al., 2004; Pomeranz et al., 2005).

PRV contains a double-stranded DNA genome of approximate 150 kbp encoding over 70 genes, surrounded by a capsid, tegument, and envelope (Pomeranz et al., 2005). It infects cells by membrane fusion. The viral glycoprotein gD mediates the binding of PRV to cells following a loose interaction between gC and the heparan sulfate on the cell surface. The binding initiates the fusion of PRV with plasma membrane mediated by the coordinated action of gB, gH, and gL. Once inside cells, the capsid and tegument proteins are transported to the nucleus via microtubules and nuclear pores. Within the nucleus, the tegument protein VP16 transactivates the transcription of the only immediate-early protein, IE180. The expressed IE180 subsequently transcriptionally activates the early genes of PRV. The early proteins of PRV can be divided into two main categories. The first category, comprising UL23, UL39/UL40, UL50, UL5, UL8, UL9/OBP, UL29/ssDNABP, UL30/DNA Pol, UL42/Pap, and UL52, are essential for nucleotide synthesis and DNA replication. The second category comprises three proteins (EP0, US1, and UL54), which are thought to act as transcription regulators, regulating the expression of genes of PRV and cells. The onset of DNA synthesis signals the start of the synthesis of main structural proteins (late protein), followed by the assembling of mature capsid and the packaging of the viral DNA (reviewed by Pomeranz et al., 2005; Nauwynck et al., 2007).

IFN-I-mediated innate immune response is the front line of host defense against viral infections (Samuel, 2001; Katze et al., 2002). Like other pathogens, viruses contain conserved molecular features of pathogens, called pathogen-associated molecular patterns (PAMPs), notably viral RNA and DNA, which can be recognized by pattern-recognition receptors (PRRs) in the host cells upon viral infection (Paludan et al., 2011). The well-known PRRs include Toll-like receptors (TLRs), retinoic acid-inducible gene-I (RIG-I)-like receptors (RLRs), nucleotide-binding oligomerization domain (NOD)-like receptors (NLRs), absent in melanoma 2 (AIM2)-like receptors (ALRs), and cytosolic DNA-sensing receptors (Brittle et al., 2004; Pomeranz et al., 2005; Ai et al., 2018; Yang et al., 2019; Liu et al., 2020). PRR engagement then activates the downstream adaptor proteins, such as stimulator of interferon genes (STING), mitochondrial antiviral signaling protein (MAVS), tumor necrosis factor receptor-associated factor (TRAFs), and Toll/IL-1 receptor domain-containing adaptor inducing IFN-β (TRIF) (Lee and Kim, 2007; Brennan and Bowie, 2010; Takeuchi and Akira, 2010; Brubaker et al., 2015; Zheng, 2021). The activated adaptor proteins subsequently induce the interferon response factors (IRFs) or/and NF-κB signaling pathways leading to the production of IFN-Is and pro-inflammatory cytokines (Hoffmann and Akira, 2013; Wu and Chen, 2014).

After secretion, type I IFN binds to its cognate receptor (IFNAR1 and IFNAR2) on the cell surface through an autocrine or paracrine fashion. In response to this binding, the members of the Janus protein tyrosine kinase family (JAKs), JAK1 and TYK2, associated with the cytoplasmic portion of the receptors, become activated and phosphorylated. The activated JAKs subsequently phosphorylate signal transducer and activator of transcription 1 (STAT1) and STAT2. The phosphorylated pSTAT1/pSTAT2, together with IFN regulatory factor 9 (IRF9), form a trimeric complex, referred to as IFN-stimulated gene factor 3 (ISGF3), and then rapidly shuttle to the nucleus, where they bind to IFN-stimulated response element (ISRE) in DNA and initiate transcription of several downstream genes called IFN-stimulated genes (ISGs)(Brennan and Bowie, 2010; Paludan et al., 2011; Brubaker et al., 2015). Many ISG products directly affect viral replication, while others modulate additional facets of innate and adaptive immune responses (Schneider et al., 2014).

As a process of coevolution, viruses have evolved various strategies to evade host innate responses. Numerous studies have indicated that alphaherpesvirus simultaneously utilizes multiple mechanisms to dismantle the host’s innate immunity, ranging from blocking PRR induced IFN-I production, antagonizing the IFN-I signaling pathway, to neutralizing the antiviral functions of ISG products. Several excellent reviews have covered this topic extensively, mainly focusing on human alphaherpesvirus (Lee et al., 2016; Su et al., 2016; Zheng, 2018). In this review, we will explore the recent reports regarding the molecular mechanisms utilized by PRV to inhibit or evade IFN-I mediated host innate immunity, with particular emphasis on those inhibiting IFN-I induced gene transcription through the JAK-STAT pathway.

## Immune Evasion Mechanisms of Pseudorabies Virus

### Evasion of the I Interferon Induction Pathway

Alphaherpesvirus can be detected by cellular PRRs localized in various places, including the cytosol, the endolysosome, and the nucleus. The detection of viral DNA by the cytosolic sensor cyclic GMP-AMP (cGAMP) synthase (cGAS) has been proved to play a central role in controlling HSV1 infection by using cGAS knockout mice. The binding of viral DNAs to cGAS has stimulated its catalytic activity, resulting in cGAMP production. cGAMP then binds and activates STING, which facilitates TANK-binding kinase 1 (TBK1) phosphorylation and activation. Activated TBK1 subsequently phosphorylates IRF3, leading to its dimerization, nuclear translocation, recruitment of the co-transcriptional activator CBP/p300, and ultimately IFN-I gene transcription (Chen et al., 2016). Viral DNA detection and the axis of STING-TBK1-IRF3 are two crucial elements in IFN-I production. They are also the main targets of alphaherpesvirus for immune evasion. In addition, NF-kB, the master transcription factor in the immune response, is often inhibited by alphaherpesvirus through various mechanisms.

**FIGURE 1:**
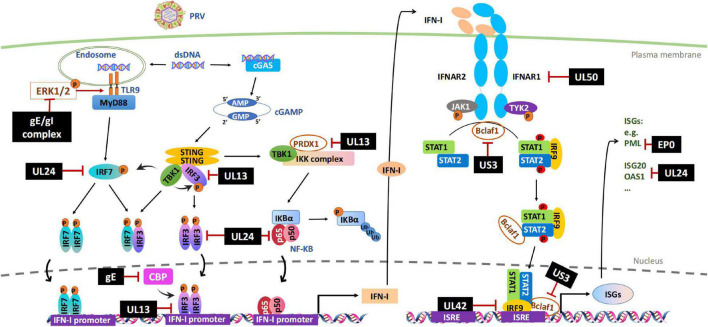
Evasion of IFN-I-mediated innate immunity by PRV. Cytosolic DNA sensors, such as cGAS and TLR9, recognize double-stranded DNA in the cytosol and trigger IFN-I production through IRFs or NF-κB signal pathways. After secretion, IFN-I binds to its cognate receptor (IFNAR1 and IFNAR2) on the cell surface and induces the transcription of antiviral factors ISGs through JAK-STAT signal pathway. PRV encoded multiple proteins can target various steps involved in this process, including hijacking DNA-sensor-mediated viral recognition and subsequent signaling, disrupting JAK-STAT signaling or inhibiting specific ISGs. The black boxes indicate the PRV proteins that are confirmed to hijack IFN-I signal pathway. P, phosphrylation; Ub, ubiquitination.

The DNA sensing pathway induced by innate immunity plays a critical role in controlling PRV infection but is antagonized by PRV infection. Accordingly, stimulation of cellular DNA sensing pathways by inducing genomic DNA damage or reducing ectonucleotide pyrophosphatase phosphodiesterase 1 (ENPP1) to increase the cellular level of cGAS can attenuate PRV infections (Wang et al., 2018; Wang J. et al., 2020). Although it is not clear whether PRV has evolved the mechanisms to avoid viral sensor detection, several studies have shown that the stimulation of the STING signaling pathway by PRV infection is dampened by multiple viral proteins, including UL13, UL24, and gE/gI (Figure 1 and Table 1).

**TABLE 1:** Summary of PRV factors antagonizing the IFN-I system.

PRV factors	Target pathway	Actions	References
gE/gI	IFN induction	TLR9 and ERK1/2 signaling might be involved	Han and Yoo, 2014
	IFN induction	Targets CBP for degradation and interrupts the enhanced IRF3-CBP assembly	Xing et al., 2013
UL13	IFN induction	Induces IRF3 ubiquitination degradation in a kinase dependent manner	Wang et al., 2018
	IFN induction	Phosphorylates IRF3 and inhibits the recruitment of phosphorylated IRF3 to the IRF3-responsive promoter	Asai et al., 2007
	IFN induction	Targets the IFN positive regulator PRDX1 for proteasomal degradation	Wang J. et al., 2020
UL24	IFN induction	Abrogates NF-κB activation by binding and degrading p65 in proteasome	Hwang et al., 2009
	IFN induction	Targets IFN7 for degradation	Wang et al., 2013
	ISGs	Suppresses the transcription of ISG20 and reduces RIG-I induced expression of OASL	Li et al., 2014; Bedford and Brindle, 2012
UL50	IFN signaling	Induces lysosomal degradation of IFNAR1	Chen et al., 2021a
UL42	IFN signaling	Competes with ISGF3 for ISRE binding to block efficient gene transcription.	Lamote et al., 2017
US3	IFN signaling	Targets the IFN positive regulator Bclaf1 for proteasome degradation	Watarai et al., 2008
EP0	ISGs	disrupts the subnuclear antiviral structure PML-NB	Piroozmand et al., 2004

*TLR9, toll-like receptor; ERK1/2, extracellular signal-regulated kinase 1/2; CBP, CREB-binding protein; IRF, interferon regulatory factor; PRDX1, cellular antioxidant enzyme peroxiredoxin 1; NF-κB, nuclear factor kappa B; ISG, Interferon-stimulated gene; RIG-I, retinoic acid-inducible gene-I; OASL, Oligoadenylate synthetases-like; IFNAR1, Interferon receptor 1; ISGF3, Interferon-stimulated gene factor 3; ISRE, Interferon-sitimulated response element; Bclaf1, Bcl-2 associated transcription factor 1; PML-NBs, Promyelocytic leukemia.*

#### UL13

UL13 is a conserved serine/threonine-protein kinase expressed in the early stages of the infection of alphaherpesviruses and directly modulates the phosphorylation of viral proteins VP11/12, ICP22, and UL49 (Tanaka et al., 2005; Asai et al., 2007; Eaton et al., 2014). It can also phosphorylate host proteins and change their functions. Several studies have demonstrated that PRV UL13 inhibits the IFN-β production by targeting IRF3 or/and other components in the pathway in a kinase-dependent manner (Bo et al., 2020; Lv et al., 2020, 2021). Lv et al. (2020) reported that UL13 inhibits the IFN-β production by targeting IRF3 for degradation. PRV with a deletion of UL13 is impaired in its ability to hinder IRF3 and IFN-β activation and has significantly less pathogenesis in mice than the wild-type PRV (Lv et al., 2020). The kinase activity sites of Lys49 and Lys387 in UL13 were found to mediate the degradation of IRF3 (Lv et al., 2020). In addition, the same group has recently reported that the cellular antioxidant enzyme peroxiredoxin 1 (PRDX1) is a positive regulator in IFN-I production, which PRV UL13 also targets for ubiquitination and degradation (Lv et al., 2021). However, it is not clear how UL13 degrades IRF3 and PRDX1. Another report also showed that PRV UL13 targets IRF3 for immune evasion but with a different mechanism. Instead of inducing IRF3 degradation, PRV UL13 inhibits cGAS-STING-mediated IFN-β production by inducing atypical IRF3 phosphorylation(s). The authors further showed that UL13 induced IRF3 phosphorylation does not inhibit IRF3 dimerization, nuclear translocation, and CBP binding but inhibits the recruitment of the activated IRF3 to the IRF3-responsive promoter and subsequent expression of ISGs induced by the cGAS–STING pathway (Bo et al., 2020).

IRF3 is an important target for herpesviral kinases for immune evasion, including UL13 and US3, which may interfere with the transcriptional activity of IRF3 at any of the following steps, including IRF3 phosphorylation by TBK1, IRF3 dimerization, nuclear localization, complex formation with CBP/p300, and binding to target gene promoters (Hwang et al., 2009; Wang et al., 2013; Bo et al., 2020). Although the kinase activity is required to disrupt most of the processes, the kinase-independent activity of UL13 is also reported, which inhibits the activated IRF3 binding with CREB-binding protein (CBP) (Hwang et al., 2009). CBP is a histone acetyltransferase and plays a key role in transcription regulation. The CBP/p300 coactivators interact with several transcription factors, including IRF3, NF-κB, STATs, and p53 (Goodman and Smolik, 2000; Bedford and Brindle, 2012). They are often targeted by viruses for host immune evasion (Xing et al., 2013; Han and Yoo, 2014; Zhang et al., 2016).

#### UL24

The UL24 of PRV is a conserved gene across many herpesviruses and is crucial for efficient viral replication (Pearson and Coen, 2002; Blakeney et al., 2005). Previous studies have demonstrated that HSV-1 UL24 inhibits DNA-sensor mediated IFN production by binding to the NF-κB subunits p65 and p50, thereby blocking their nuclear translocations (Xu et al., 2017). Several recent studies have shown that PRV UL24 also contributes to PRV-mediated evasion of IFN and other innate immunity pathways. Wang T.Y. et al. (2020) reported that PRV UL24 protein abrogated tumor necrosis factor-alpha (TNF-α)-mediated NF-κB activation by selectively interacting with p65 and promoting it for proteasomal degradation. Liu et al. (2021) found that PRV UL24 efficiently inhibited cGAS-STING mediated IFN production by interacting with and degrading interferon regulatory factor 7 (IRF7) through the proteasome pathway. Chen et al. (2021a) found that PRV UL24 protein impaired RIG-I signaling and reduced RIG-I induced expression of endogenous ISGs in an IRF3-dependent manner.

#### gE/gI

The PRV transmembrane glycoprotein gE and gI form a heterodimer. Together they are involved in virulence and are required for anterograde neuronal transport of viral particles. It has been that gE/gI is involved in IFN-I production in plasmacytoid dendritic cells (pDC). pDC plays a central role in the antiviral immune response by producing massive amounts of IFN-I after sensing viruses through endosomal toll-like receptors 7 and 9 (TLR7/9) (Watarai et al., 2008). By comparing the live attenuated PRV Bartha vaccine strain and the wild-type strains of PRV, Lamote et al. (2017) found that the PRV Bartha vaccine strain triggered a much stronger IFN-I response in porcine pDC. They further showed that the absence of viral gE/gI glycoprotein complex in Bartha contributes to the increased IFN-I response observed. Although the exact mechanism of how the viral gE/gI complex suppresses IFN-I production by pDC is unclear, it might involve the extracellular signal-regulated kinase 1/2 (ERK1/2), which is one of the most well-characterized members of the mitogen-activated protein kinase family and regulates a range of processes, from metabolism, motility, and inflammation, to cell death and survival, as the absence of gE leads to enhanced ERK1/2 phosphorylation in pDC, which correlates with higher IFN-I production.

A recent study reported that PRV gE is involved in counteracting cGAS/STING-mediated IFN production through degrading CBP (Lu et al., 2021). Lu et al. (2021) found that ectopic expression of PRV gE decreased cGAS/STING-mediated IFN-β promoter activity and the level of mRNA expression. Further investigation revealed that gE was located on the nuclear membrane inducing CBP degradation, resulting in IRF3 being unable to bind CBP leading to inhibition of IFN-β production.

### Evasion of the I Interferon Signaling Pathway

Inhibiting the JAK-STAT pathway by viruses is a key step in stopping ISGs production and further amplifying IFN-I. It has been known that PRV infection inhibits IFN induced STATs phosphorylation and ISG transcriptions (Brukman and Enquist, 2006b). Recent studies have indicated that PRV has evolved multiple strategies to disrupt the JAK-STAT signaling pathway, ranging from inducing the degradation of critical signaling molecules, including IFNAR1 and JAKs, to blocking the binding of ISGF3 to ISG promoters (Zhang et al., 2017, 2021; Qin et al., 2019; Yin et al., 2021). The involved viral proteins include but are probably not limited to UL50, UL42, and US3 (Figure 1 and Table 1).

#### UL50

PRV UL50 is a deoxyuridine triphosphate nucleotidohydrolase (dUTPase) catalyzing dUTP hydrolysis into dUMP and inorganic pyrophosphate. This reaction provides dUMP, the precursor for dTTP biosynthesis (Harris et al., 1999), and is critical for viral DNA replication in certain cell types. A study in our laboratory (Zhang et al., 2017) found that the UL50 proteins of both PRV and HSV-1 possess the ability to suppress IFN-mediated JAK-STAT signaling, but interestingly, this activity is independent of the dUTPase enzymatic activity. Of note, the activity of HSV-1 UL50 is much weaker than that of PRV UL50. Mechanistically, UL50 impeded type I IFN-induced STAT1 phosphorylation, likely by accelerating lysosomal degradation of IFNAR1. Compared with WT PRV, the UL50 deletion virus is more sensitive to IFN-I mediated viral suppression (Zhang et al., 2017). Interestingly, the sequence in PRV UL50 that mediates the IFN inhibition is nearly absent in the cellular dUTPase but is present in other herpesviral dUTPases shown to inhibit IFN-signaling, including HSV-1 UL50 as well as γ-herpesviruses KSHV and MHV68 ORF54 (Leang et al., 2011; Madrid and Ganem, 2012), suggesting that during herpesvirus evolution, the virus may have acquired a specific sequence in its dUTPase to improve viral replication and fitness.

#### UL42

UL42 is a highly conserved DNA polymerase processivity factor in alphaherpesviruses, important for virus DNA replication (Digard et al., 1993). The functional analysis of HSV-1 UL42 has shown that it performs three primary biochemical functions, including binding to DNA, stably associating with virus DNA polymerase UL30, and increasing the length of DNA chains synthesized by UL30 (Gottlieb et al., 1990; Digard et al., 1993; Thornton et al., 2000). Interestingly, early studies showed that about half of the UL42 protein was bound to UL30, while the rest was free from this complex, likely functioning beyond DNA replication (Gottlieb et al., 1990; Chapon et al., 2019). Recently, our group found that UL42 proteins of PRV and HSV-1 could disrupt IFN-mediated activation of JAK-STAT signaling, leading to a decreased transcription and expression of ISGs, which contributes to PRV infection mediated IFN-I immune evasion. Further investigation demonstrated that UL42 directly interacted with ISRE and competitively interfered with ISGF3 binding to ISRE for efficient gene transcription. The four conserved DNA-binding sites of UL42 are essential for this activity (Zhang et al., 2021). Zhang et al. (2013) and Chapon et al. (2019) have previously reported that HSV-1 UL42 could suppress IFN-β transcriptional activation by interfering with IRF-3 or NF-κB signaling, but the specific mechanisms are not very clear. The IRF3-binding element (IRE) of the IFN-β gene and ISRE of ISGs have a similar and overlapping consensus-binding sequence 5′-GAAANNGAAACT-3′ (Ourthiague et al., 2015). Therefore, perhaps UL42 might also be able to bind to IRE and inhibit IFN-β transcription competitively. Nevertheless, these findings indicate that in addition to being an essential accessory factor in viral DNA replication, UL42 of alphaherpesviruses inhibits the host’s innate immune response, which is a good target for anti-herpesvirus drug development.

#### US3

US3 is a conserved Ser/Thr kinase encoded by every alphaherpesvirus identified thus far (Deruelle and Favoreel, 2011), involving the pathogenicity of the viruses *in vivo* (Wagenaar et al., 1995; Reynolds et al., 2002). As a viral kinase, US3 expression also impacts host antiviral responses in many aspects. For instance, US3 of HSV-1 and PRV has been reported to prevent host cells from apoptosis (Leopardi et al., 1997; Benetti and Roizman, 2007; Chang et al., 2013), disrupt the antiviral subnuclear structure promyelocytic leukemia nuclear body (PML-NB) (Jung et al., 2011), down-regulate major histocompatibility complex (MHC) class I surface expression (Rao et al., 2011) and inhibit IFN production (Piroozmand et al., 2004; Liang and Roizman, 2008; Wang et al., 2013, 2014). A study performed in our laboratory showed that US3 of PRV and HSV-1 inhibits the IFN induced antiviral gene transcription by inducing the degradation of a positive regulator of IFN signaling, Bclaf1 (Bcl-2 associated transcription factor 1) (Qin et al., 2019). Bclaf1, on the one hand, regulates IFN induced STAT1/STAT2 phosphorylation with an unknown mechanism; on the other hand, it enhances the recruitment of ISGF3 complex to the promoter of the ISGs by forming an ISGF3-Bclaf1-ISRE complex (Qin et al., 2019). During PRV infection, Bclaf1 is phosphorylated by US3, leading to its ubiquitination and degradation, which facilitates PRV replication in the presence of IFN (Qin et al., 2019). The detailed mechanism of how US3 degrades Bclaf1 is still under investigation.

### Inhibition of IFN-Stimulated Gene Expression and Function

IFN-Is trigger the induction of numerous ISGs, including promyelocytic leukemia (PML) protein, interferon-stimulated gene 20 (ISG20), 2′-5′ oligoadenylate synthetase (OAS), interferon-stimulated gene 15 (ISG15), and cholesterol 25-hydroxylase (CH25H). These ISGs promote an antiviral state that limits PRV replication and targets viral immune evasion (Schneider et al., 2014). During the past few years, PRV EP0 and UL24 have been shown to impair the expression and function of ISGs (Figure 1).

#### EP0

EP0 is a PRV early gene product possessing a RING-finger ubiquitin ligase activity. It is known that EP0 can disrupt PML-NBs by inducing PML protein ubiquitination and degradation. PML-NBs are a sub-nuclear structure associated with several antiviral proteins, including Sp100, Daxx, and ATRX. They can inhibit viral replication through multiple mechanisms such as suppressing viral gene transcription and inducing epigenetic silence of viral genomes (Everett and Chelbi-Alix, 2007; Geoffroy and Chelbi-Alix, 2011; Alandijany et al., 2018). We have recently reported that swine PML-NBs also inhibit PRV replication (Yu et al., 2020). PML is IFN induced protein. In response to IFN treatment, the level of PML protein and the number and sizes of PML-NBs are all increased (Brukman and Enquist, 2006a). Reported that EP0 could counteract the IFN-mediated antiviral response in primary cells isolated from the natural host of PRV. We also found that the EP0-deletion PRV strain (PRV-EP0 KO) was more sensitive to IFN treatment than the PRV wild-type strain (PRV-WT). One likely mechanism underlying this observation is that EP0 induces PML-NB disruption and degradation, impairs PML-NB mediated antiviral functions. Indeed, in PML knockout cells, the difference in the sensitivity to IFN between PRV-EP0 and PRV-WT is reduced. However, PRV-EP0 is still more sensitive to IFN treatment, indicating other mechanisms are also involved (Yu et al., 2020).

EP0 is a homolog of HSV-1 ICP0. They both contain a conserved RING-finger region but differ tremendously in the sequence of the RING domain and temporal expression (Everett et al., 2010; Li et al., 2014). ICP0 is an immediate-early gene and known to play a critical role in innate immunity evasion, mostly by degrading a wide range of proteins involved in intrinsic and innate immunity, such as PML, DNA-PKcs, and IFI16 (Lees-Miller et al., 1996; Orzalli et al., 2012; Jan Fada et al., 2020). In comparison, the study on EP0 mediated host protein degradation is very limited. EP0 is an early gene but is present in the tegument of PRV virions. Thus, EP0 may have an opportunity to exert its functions soon after PRV enters host cells as its HSV-1 counterpart. However, whether EP0 is functionally equivalent to ICP0 in immune evasion is unclear but warrants further investigation.

#### UL24

Recent studies have demonstrated that the transcription and expression of ISG20 and OASL are antagonized by UL24 during PRV infection, underscoring the importance of UL24 in immune evasion.

ISG20 modulates PRV replication by enhancing IFN signaling. Chen et al. (2021b) reported that ectopic expression of ISG20 upregulated IFN-β expression and enhanced IFN downstream signaling during PRV infection, leading to reduced PRV proliferation. On the contrary, PRV UL24 expression suppressed the transcription of ISG20 and thus antagonizing its antiviral effect. Additionally, Chen et al. (2021b) found that the mRNA levels of ISG20 were higher in UL24-null PRV infected cells than those in WT PRV infected cells, indicating that UL24 plays an important role in suppressing ISG20 and promoting PRV proliferation.

The OAS family belongs to the ISG family and produces 2′–5′ oligoadenylates, which trigger RNA degradation by activating RNase L (Kristiansen et al., 2011). The OAS family consists of four distinct OAS isoforms, and OASL is one of them. Chen et al. (2021a) found that OASL regulates PRV proliferation by enhancing RIG-I signaling and boosting RIG-I -mediated IFN expression. However, PRV infection decreased the expression of OASL at both the mRNA and protein levels. Furthermore, PRV UL24 protein impaired RIG-I signaling and reduced RIG-I induced expression of endogenous OASL in an IRF3-dependent manner, thereby antagonizing the OASL antiviral effect (Chen et al., 2021a).

## Future Perspective

Although hosts have evolved powerful innate immune mechanisms, mainly mediated by IFN-Is, in response to virus invasion, PRV has evolved strategies to hijack host immune responses for viral replication and the establishment of persistent infection. PRV uses multiple viral proteins to curtail various components in the IFN response network by either degrading them, reducing their mRNA accumulations or interfering with their functions. The multi-facet efforts used by PRV to disrupt the IFN system probably are to ensure that the antiviral effect of IFN is effectively blocked so that PRV can successfully establish persistent infection.

As a distant relative of human alphaherpesviruses, PRV shares considerable functional gene homology with human herpesviruses and possesses a broader host range, making it an ideal model to examine host-pathogen interactions, especially for the studies of different outcomes of PRV infection in the natural and non-natural hosts. To understand host-viral interaction with the perspective of human and animal herpesviral immune evasion, more comparative studies should be investigated, which could offer useful references for a deep understanding of the immune evasion mechanism of alphaherpesviruses. Comparative studies would help to identify key molecules and pathways targeted by all alphaherpesviruses and uncover the mechanisms unique to PRV that human alphaherpesviruses might potentially adopt in the future. In addition, there is a need to understand the differential outcome of PRV infection of pigs and other species from the point of the innate antiviral immunity and inflammatory responses and identify key elements that spare adult pigs from the infection-induced mortality. Lastly, PRV infection of its natural host is an ideal model to examine cell type-specific response to PRV infection *in vivo*. It is interesting to determine whether the differential control of PRV in different cell types contributes to its neuronal spread and establishing latency *in vivo*.

Finally, as a veterinary pathogen, PRV was once under control due to the vaccination in China. However, the emergence of antigenically different PRV variants leads to the re-transmission and epidemic of PR, even becoming a potential threat to humans. These emerged new strains exhibit much stronger pathogenicity. It remains to be determined whether these new strains have evolved new strategies to evade innate immunity and whether these strategies contribute to enhanced pathogenicity. Understanding these questions will help to design better vaccines for PRV controls.

In conclusion, PRV is unique in many regards. Future PRV research will promise significant contributions to comparative virology, neurobiology, and cellular biology.

## Author Contributions

RZ drafted, wrote the manuscript, and designed the artwork. JT revised the manuscript and provided valuable suggestions. Both authors read and approved the final manuscript.

## Conflict of Interest

The authors declare that the research was conducted in the absence of any commercial or financial relationships that could be construed as a potential conflict of interest.

## Publisher’s Note

All claims expressed in this article are solely those of the authors and do not necessarily represent those of their affiliated organizations, or those of the publisher, the editors and the reviewers. Any product that may be evaluated in this article, or claim that may be made by its manufacturer, is not guaranteed or endorsed by the publisher.
